# Long-acting muscarinic antagonist and long-acting β_2_-agonist combination for the treatment of maintenance therapy–naïve patients with chronic obstructive pulmonary disease: a narrative review

**DOI:** 10.1177/17534666241279115

**Published:** 2024-10-01

**Authors:** Roland Buhl, Marc Miravitlles, Antonio Anzueto, Stephen Brunton

**Affiliations:** Pulmonary Department, Johannes Gutenberg University Hospital, Mainz, Germany; Pneumology Department, Hospital Universitari Vall d’Hebron/Vall d’Hebron Institut de Recerca (VHIR), Vall d’Hebron Barcelona Hospital Campus, Barcelona, Spain; The University of Texas Health Science Center at San Antonio, San Antonio, TX 78229-3901, USA; South Texas Veterans Health Care System, San Antonio, TX 78229-3901, USA; Primary Care Respiratory Group, Winnsboro, SC, USA

**Keywords:** chronic obstructive pulmonary disease, Global Initiative for Obstructive Lung Disease, long-acting β_2_-agonist, long-acting muscarinic antagonist, maintenance therapy–naïve patients, narrative review

## Abstract

Chronic obstructive pulmonary disease (COPD) is a leading cause of morbidity and mortality worldwide. Faster lung function impairment occurs earlier in the disease, particularly in mild-to-moderate COPD, highlighting the need for early and effective targeted interventions. The Global Initiative for Chronic Obstructive Lung Disease (GOLD) 2024 report recommends initial pharmacologic treatment with a long-acting muscarinic antagonist (LAMA) and long-acting β_2_-agonist (LABA) combination in group B (0 or 1 moderate exacerbation not leading to hospitalization, modified Medical Research Council score of ⩾2, and COPD Assessment Test™ score of ⩾10) and E (⩾2 moderate exacerbations or ⩾1 exacerbation leading to hospitalization and blood eosinophil count <300 cells/µL) patients. In randomized controlled trials (RCTs), LAMA/LABA combination therapy improved lung function, St. George’s Respiratory Questionnaire (SGRQ) total score, and Transitional Dyspnea Index (TDI) focal score and reduced the use of rescue medications, exacerbation risk, and risk of first clinically important deterioration (CID), compared with LAMA or LABA monotherapy. However, there is limited evidence regarding the efficacy and safety of LAMA/LABA combination therapy versus LAMA or LABA monotherapy in maintenance therapy–naïve patients. This review discusses the rationale for the early initiation of LAMA/LABA combination therapy in maintenance therapy–naïve patients with COPD. In post hoc analyses of pooled data from RCTs, compared with LAMA or LABA monotherapy, LAMA/LABA combination therapy improved lung function and quality of life and reduced COPD symptoms, risk of first moderate/severe exacerbation, risk of first CID, and use of rescue medication, with no new safety signals. In a real-world study, patients initiating LAMA/LABA had significantly reduced risk of COPD-related inpatient admissions and rate of on-treatment COPD-related inpatient admissions over 12 months than those initiating LAMA. Consequently, LAMA/LABA combination therapy could be considered the treatment of choice in maintenance therapy–naïve patients with COPD, as recommended by the GOLD 2024 report.

## Plain language summary

Long-acting bronchodilator combination therapy for the treatment of maintenance therapy–naïve patients with chronic obstructive pulmonary disease

Chronic obstructive pulmonary disease (COPD) is a common lung disease that makes it hard to breathe and is a leading cause of death and disability worldwide. This disease tends to worsen lung function from an early stage, especially in people who only have mild or moderate symptoms. To help stop the loss of lung function and maintain the quality of life for patients with COPD, two main types of long-lasting inhaler medications are used: one type focuses on relaxing the muscles around the airways, and the other type helps open the airways making it easier to breathe. Some medications combine these two types of action and are approved for long-term management of COPD. However, there is not much information on the effectiveness and safety of these combination medications in patients who have never taken long-lasting COPD medication before. Current health guidelines suggest starting these combination medications in patients who are likely to see their symptoms get worse quickly, and who do not have a high level of a specific type of white blood cell. In this review, we discuss the evidence for starting these combination treatments early in patients who have never used long-lasting COPD medications before. There is no strong evidence yet that shows starting treatment early benefits patients with newly diagnosed COPD. However, about 30% of patients in clinical trials designed to study the effectiveness of these combination medications, had never received any long-lasting treatment before. After-the-fact analyses of these patients showed that these combination medications could reduce symptoms such as breathlessness, improve lung function, enhance quality of life, lessen the need for emergency medications, and decrease the risk of severe symptom flare-ups. Overall, the evidence supports using these combination inhaler medications as the first choice of treatment for patients with moderate COPD symptoms who have not previously been treated with long-lasting inhalers.

## Introduction

Chronic obstructive pulmonary disease (COPD) is a major cause of morbidity and mortality worldwide, with extensive healthcare and economic costs.^1^ The main treatment goals for stable COPD are the reduction of both symptoms and future exacerbation risk.^2^ Additionally, studies have suggested that faster lung function impairment occurs earlier in the disease course, particularly in mild-to-moderate COPD.^3–5^ In a predictive modeling study assessing the impact of initiating pharmacotherapy earlier versus later on long-term COPD progression, the preservation of lung function was higher when treatment was initiated earlier than later during the disease course.^6^ In clinical practice, the majority of patients present with moderate COPD and have a reduced quality of life (QoL).^7^ These findings highlight the need for early and effective targeted interventions to prevent the loss of lung function and preserve QoL in patients with COPD.

Long-acting muscarinic antagonists (LAMAs) and long-acting β_2_-agonists (LABAs) are the mainstay of COPD management because of their synergistic effects and long duration of action.^2,8^ LABAs stimulate β_2_-adrenergic receptors on airway smooth muscles, thereby producing functional antagonism to bronchoconstriction.^2,9^ LABAs such as formoterol and salmeterol have a duration of action of 12 h, and some other LABAs (also known as ultra-LABAs; e.g., olodaterol, vilanterol, and indacaterol) have a duration of action of 24 h.^2,9^ LAMAs block the bronchoconstrictor effects of acetylcholine on M3 muscarinic receptors expressed on airway smooth muscles and have a duration of action of 12–24 h (e.g., aclidinium, glycopyrronium, umeclidinium, and tiotropium).^2,9^ LABAs and LAMAs significantly improve lung function, dyspnea, and health status, and decrease exacerbation rates.^2^ Compared with LABAs, LAMAs have a greater effect on exacerbation reduction and decrease hospitalizations.^2^ Considering the different but complementary mechanisms of action, combining a LAMA with a LABA helps achieve better clinical outcomes in patients with COPD who are not effectively controlled with a single long-acting bronchodilator.^10,11^ When initiating treatment with long-acting bronchodilators, a combination of LAMA and LABA is the preferred choice.^2^ Table 1 presents the single-inhaler fixed-dose combinations (FDCs) of LAMA/LABA that have been approved in the United States (US) for the maintenance treatment of patients with COPD.^12–16^

**Table 1.** Overview of single-inhaler LAMA/LABA fixed-dose combination therapy for maintenance treatment of patients with COPD.

Generic drug name (brand name)	Initial US approval	Dosage form and strength	Dosage and administration	Inhaler type^2^	Duration of action^2^
Tiotropium bromide and olodaterol (STIOLTO^®^ RESPIMAT^®^)^12^	2015	Each actuation delivers 2.5 µg of tiotropium (equivalent to 3.124 µg of tiotropium bromide monohydrate), and 2.5 µg of olodaterol (equivalent to 2.736 µg of olodaterol hydrochloride). Two actuations equal one dose	Two inhalations once daily	Soft mist inhaler	24 h
Umeclidinium and vilanterol (ANORO ELLIPTA)^13^	2013	Inhalation powder: 62.5 µg of umeclidinium and 25 µg of vilanterol (62.5/25 µg) per actuation	One actuation administered once daily by oral inhalation	Dry powder inhaler	24 h
Glycopyrronium and indacaterol (UTIBRON^®^ NEOHALER^®^)^14^	2015	Inhalation powder: 27.5 µg of indacaterol and 15.6 µg of glycopyrronium inhalation powder per capsule for use with the NEOHALER device	Twice daily by oral inhalation	Dry powder inhaler	12–24 h
Glycopyrronium and formoterol fumarate (BEVESPI AEROSPHERE^®^)^15^	2016	Pressurized metered dose inhaler containing a combination of glycopyrronium (9 µg) and formoterol fumarate (4.8 µg) per inhalation	Two inhalations twice daily by oral inhalation	Pressurized metered dose inhaler	12 h
Aclidinium bromide and formoterol fumarate (DUAKLIR^®^ PRESSAIR^®^)^16^	2019	Breath-actuated multidose dry powder inhaler metering 400 µg of aclidinium bromide and 12 µg of formoterol fumarate per actuation	400 µg/12 µg, twice daily	Dry powder inhaler	12 h

COPD, chronic obstructive pulmonary disease; LABA, long-acting β_2_-agonist; LAMA, long-acting muscarinic antagonist; US, United States.

There is no high-quality evidence, such as that from randomized controlled trials (RCTs), to support initial pharmacological treatment strategies in newly diagnosed patients with COPD.^2^ Each pharmacological treatment regimen should be individualized and guided by symptom severity; exacerbation risk; side effects; comorbidities; drug availability and cost; and the patient’s response, preference, and ability to use various drug delivery devices.^2^ The Global Initiative for Chronic Obstructive Lung Disease (GOLD) 2024 report,^2^ 2020 American Thoracic Society Clinical Practice Guideline,^8^ 2021 Spanish guidelines for COPD,^17^ and 2018 National Institute for Health and Care Excellence guidelines^18^ endorse the use of LAMA/LABA as an initial pharmacologic treatment in patients who have a high risk of exacerbation but do not have eosinophilia. Differing slightly, the 2023 Canadian Thoracic Society (CTS) Guideline on Pharmacotherapy in Patients With Stable COPD recommends the use of LAMA/LABA as an initial treatment in patients with moderate-to-severe disease at *low risk* of exacerbations; however, it also suggests the use of LAMA/LABA/ICS for those at *high risk* of exacerbation regardless of eosinophil levels (Table 2).^19^

**Table 2.** Initial pharmacologic treatment in patients with COPD.

GOLD 2024^2^	CTS 2023^19^	2020 ATS Clinical practice guidelines^8^	2021 Spanish COPD guidelines^17^	2018 NICE guidelines^18^
• LAMA/LABA is recommended for patients in: ○ Group B (0 or 1 moderate exacerbations [not leading to hospital admission], modified Medical Research Council score of ⩾2, and COPD Assessment Test™ score of ⩾10); and ○ Group E (⩾2 moderate exacerbations or ⩾1 leading to hospitalization and blood eosinophil count < 300 cells/µL) categories • ICS is recommended for Group E patients with blood eosinophil count ⩾300 cells/µL	• LAMA/LABA is recommended for patients with: ○ stable COPD, low risk of exacerbations, a moderate-to-high symptom burden and/or health status impairment (CAT ⩾ 10, mMRC ⩾ 2), and impaired lung function (FEV_1_ < 80% predicted) • LAMA/LABA/ICS is recommended for patients with: ○ stable COPD, high risk of exacerbations, a moderate-to-high symptom burden and/or health status impairment (CAT ⩾ 10, mMRC ⩾ 2), and impaired lung function (FEV_1_ < 80% predicted)	• No recommendations for the initial treatment of patients with COPD • Strong recommendation for the use of LAMA/LABA over LABA or LAMA monotherapy in patients with COPD and dyspnea or exercise intolerance • Conditional recommendation for the use of triple therapy (LAMA/LABA/ICS) over LAMA/LABA in patients with COPD and dyspnea or exercise intolerance who have experienced ⩾1 exacerbation in the past year requiring antibiotics or oral steroids or hospitalization	• Recommend LAMA monotherapy as initial treatment in patients with COPD with low exacerbation risk, along with subsequent treatment with LAMA/LABA, if the patients experience an exacerbation • In patients with COPD who are at high exacerbation risk but without eosinophilia, LAMA/LABA is recommended as the initial treatment • In patients with eosinophilia, LABA/ICS is recommended as initial treatment followed by escalation to triple therapy (LAMA/LABA/ICS)	• Recommend LAMA/LABA for patients who have spirometrically confirmed COPD and do not have features suggestive of asthma/steroid responsiveness • LAMA/LABA is also recommended for patients who remain breathless or experience exacerbations despite having used or been offered treatment for tobacco dependence for patients who smoke and optimized non-pharmacological management, relevant vaccinations, and use of a short-acting bronchodilator

ATS, American Thoracic Society; CAT, COPD Assessment Test; COPD, chronic obstructive pulmonary disease; GOLD, Global initiative for chronic Obstructive Lung Disease; ICS, inhaled corticosteroid; LABA, long-acting β_2_-agonist; LAMA, long-acting muscarinic antagonist; NICE, National Institute for Health and Care Excellence.

This review discusses the role of LAMA/LABA combination treatment as first-line maintenance therapy in maintenance therapy–naïve patients with COPD as an alternative to other therapies (including monotherapy) in reducing exacerbation risk, improving disease management, preserving lung function, and enhancing QoL.

## Clinical trials of LAMA/LABA combination therapy in maintenance therapy–naïve patients with COPD

Evidence for the use of LAMA/LABA combination therapy in maintenance therapy–naïve patients is mostly based on post hoc analyses of pooled data from RCTs.^20–28^ Thus, we provide a brief overview of these RCTs before describing the results for the maintenance therapy–naïve subgroup. Considering that this is a narrative review, the list of included articles may not be exhaustive and is based on the authors’ experience in the management of patients with COPD.

### Tiotropium/olodaterol

TOnado 1 (NCT01431274) and TOnado 2 (NCT01431287) were two replicate, global, and phase III trials that aimed to assess the efficacy and safety of once-daily treatment with orally inhaled tiotropium+olodaterol FDC 5/5 µg or 2.5/5 µg delivered *through* the Respimat^®^ soft mist inhaler, compared with their individual mono-components, in patients with moderate-to-very severe COPD (GOLD stage 2–4) over 52 weeks.^29^ Significant improvements were observed in lung function (forced expiratory volume in 1 s (FEV_1_) area under the curve from 0 to 3 h (AUC_0–3_) and trough FEV_1_ response) and health-related QoL (St. George’s Respiratory Questionnaire [SGRQ] total score); additionally, the use of rescue medications and exacerbation rates were reduced with once-daily tiotropium+olodaterol FDC versus mono-components over 1 year in patients with moderate-to-very severe COPD.^29^ OTEMTO 1 (NCT01964352) and OTEMTO 2 (NCT02006732) were two replicate, double-blind, parallel-group, placebo-controlled trials in which patients were randomized to receive tiotropium+olodaterol 5/5 μg or 2.5/5 μg, tiotropium 5 μg, or placebo for 12 weeks through the Respimat^®^ inhaler.^30^ Tiotropium/olodaterol improved lung function and QoL compared with placebo and tiotropium.^30^

### Umeclidinium/vilanterol

Early MAXimisation of bronchodilation for improving COPD stability (EMAX) was a 24-week, multicenter, randomized, double-blind, double-dummy, three-arm, parallel-group trial that investigated the efficacy and safety of umeclidinium/vilanterol versus umeclidinium and salmeterol monotherapies in patients with COPD at low exacerbation risk who were not receiving inhaled corticosteroids (ICS).^31^ Umeclidinium/vilanterol consistently provided early and sustained improvements in lung function and symptoms and reduced the risk of deterioration/treatment failure versus umeclidinium or salmeterol in symptomatic patients with low exacerbation risk of not receiving ICS.^31^ Two large, 24-week, randomized, double-blind, placebo-controlled trials (NCT01313637 and NCT01313650) with replicate design compared the efficacy and safety of umeclidinium/vilanterol (NCT01313637: 125/25 µg and NCT01313650: 62.5/25 µg) versus placebo, umeclidinium (NCT01313650), and vilanterol monotherapy.^32,33^ Both these RCTs reported that once-daily umeclidinium/vilanterol was well tolerated and provided greater improvements in lung function, health status, and dyspnea scores than monotherapy and placebo over 24 weeks.^32,33^ Three multicenter, randomized, 24-week, parallel-group blinded trials (NCT01777334, NCT01316913, and NCT01316900) compared the efficacy and safety of umeclidinium/vilanterol combination (62.5/25 µg in all three trials and 125/25 µg in NCT01316913 and NCT01316900) versus vilanterol 25 µg (NCT01316900), umeclidinium 125 µg (NCT01316913), or tiotropium (18 µg through the HandiHaler^®^).^34,35^ Umeclidinium/vilanterol improved trough FEV_1_ compared with its mono-components and tiotropium in all three trials.^34,35^

### Glycopyrronium/indacaterol

SHINE (NCT01202188) was a 26-week, multicenter, double-blind, parallel-group, placebo- and active-controlled study that randomized patients to receive glycopyrronium/indacaterol 50/110 μg once daily (QD), indacaterol 150 μg QD, glycopyrronium 50 μg QD, open-label tiotropium 18 μg QD, or placebo.^36^ Glycopyrronium/indacaterol demonstrated superior and clinically meaningful outcomes versus placebo and superiority versus treatment with a single bronchodilator (LAMA or LABA), with a safety and tolerability profile similar to that seen with placebo.^36^ SPARK (NCT01120691) was a 64-week, multicenter, double-blind, parallel-group study that randomized patients to once-daily treatment with FDC of glycopyrronium/indacaterol 50/110 μg, glycopyrronium 50 μg, or open-label tiotropium 18 μg (HandiHaler).^37^ Compared with glycopyrronium monotherapy, glycopyrronium/indacaterol combination therapy was superior in preventing moderate-to-severe COPD exacerbations, with concomitant improvements in lung function and health status.^37^ ARISE (NCT01285492) was a 52-week, multicenter, open-label, parallel-group, active-controlled study that evaluated the efficacy and safety of glycopyrronium/indacaterol in Japanese patients with moderate-to-severe COPD.^38,39^ Compared with tiotropium (18 µg QD delivered via the HandiHaler^®^ device), glycopyrronium/indacaterol increased predose FEV_1_ and inspiratory capacity, reduced the rescue medication use and improved the SGRQ score.^38^ In a pooled data analysis of Japanese patients from the SHINE and ARISE studies, glycopyrronium/indacaterol significantly improved predose FEV_1_, FEV_1_ at 30 min and 60 min postdose, SGRQ total score, and rescue medication use compared with tiotropium.^39^

### Glycopyrronium/formoterol

PINNACLE-1 (NCT01854645), PINNACLE-2 (NCT01854658), and PINNACLE-4 (NCT02343458) were randomized, double-blind, parallel-group, placebo-controlled studies that compared the efficacy and safety of glycopyrronium/formoterol (18/9.6 µg) with its mono-components (glycopyrronium 18 µg and formoterol 9.6 µg) and placebo.^40,41^ Glycopyrronium/formoterol demonstrated superiority over placebo and the mono-components in improving lung function and patient-reported outcomes (PROs) and was well tolerated.^40,41^

### Aclidinium/formoterol

The ACLIFORM-COPD study (NCT01462942) was a 24-week, double-blind, randomized, parallel-group active- and placebo-controlled, multicenter study that assessed the efficacy and safety of aclidinium/formoterol versus monotherapy and placebo.^42^ Aclidinium/formoterol significantly improved bronchodilation versus monotherapy and dyspnea versus placebo, without any increased safety risk.^42^ AUGMENT-COPD (NCT01437397) was a 24-week double-blind study wherein 1692 patients with stable COPD were randomized to twice-daily treatment with FDC aclidinium 400 μg/formoterol 12 μg, FDC aclidinium 400 μg/formoterol 6 μg, aclidinium 400 μg, formoterol 12 μg, or placebo administered using a multidose dry powder inhaler (Genuair^®^/Pressair^®^).^43^ Aclidinium/formoterol (400/12 µg) provided rapid and sustained bronchodilation that was greater than either monotherapy bronchodilator and was well tolerated.^43^

While there are many trials analyzing the effectiveness of dual bronchodilation with LAMA/LABA compared to monotherapy with a LAMA or LABA, there are few head-to-head studies of different dual bronchodilator combinations. Maltais et al.^44^ and Feldman et al.^45^ are two studies that do report differences between combinations. A systematic review of the literature by Hurst et al.,^46^ however, noted the differences in patient population and design in available head-to-head studies and concluded that currently available (time of publication 2020) LAMA/LABA therapies have comparable efficacy and safety. We believe this is further evidence that each pharmacological treatment regimen should be individualized toward the patient being treated.

## LAMA/LABA combination therapy versus active monotherapy in maintenance therapy–naïve patients: Evidence from RCTs

The efficacy and safety of single-inhaler LAMA/LABA therapy have been compared with those of placebo and mono-components (LAMA or LABA) in post hoc analyses of pooled data from RCTs of LAMA/LABA maintenance therapy in patients with COPD^20–28^ and one prespecified analysis of an RCT.^47^ Maintenance therapy–naïve patients constituted approximately 30% of the overall patients included in the RCTs.^23,24,47^ Compared with the overall RCT population or maintenance therapy subgroup, patients in the maintenance therapy–naïve subgroup were slightly younger,^27,47^ were female,^47^ and had a higher proportion of current smokers,^24,27,47^ moderate COPD (GOLD stage 2),^20,24^ better lung function (higher baseline FEV_1_),^27,47^ and worse health status (higher mean COPD Assessment Test [CAT] score, Evaluating Respiratory Symptoms in COPD [E-RS COPD], and SGRQ total score).^47^ Additionally, maintenance therapy–naïve patients had a greater mean daily albuterol use^47^ and fewer moderate exacerbations in the previous year.^47^ For this review, we compared the efficacy and safety of single-inhaler LAMA/LABA versus active monotherapy on lung function, PROs, rescue medication-free days, exacerbations, clinically important deterioration (CID; a composite endpoint), and safety.

### Lung function

In a post hoc analysis of pooled data from TOnado 1 and 2, FEV_1_ responders (⩾100 mL improvement from baseline) were 29.4% and 44.5% in the tiotropium/olodaterol and tiotropium groups, respectively.^28^ In a post hoc analysis of pooled data from TOnado 1 and 2 and OTEMTO 1 and 2 in maintenance therapy–naïve patients with COPD, the increase in trough FEV_1_ from baseline at week 12 was significantly greater with tiotropium/olodaterol *versus* tiotropium (mean treatment difference ± standard error (SE): 0.056 ± 0.012 L; 95% confidence interval [CI]: 0.033–0.079; *p* < 0.0001).^20^ Compared with tiotropium monotherapy, tiotropium/olodaterol significantly improved trough FEV_1_ in patients with moderate (mean adjusted treatment difference: 56 mL; 95% CI: 25–87; *p* = 0.0004) and severe (mean adjusted treatment difference: 51 mL; 95% CI: 11–91; *p* = 0.0122) COPD, irrespective of baseline SGRQ or baseline dyspnea index (BDI) scores.^20^ In a subgroup analysis of the OTEMTO studies based on treatment history, treatment difference in FEV_1_ AUC_0–3_ was significantly higher with tiotropium/olodaterol than with tiotropium monotherapy.^21^ In another post hoc analysis of the TOnado 1 and 2 studies, the effects of two doses of tiotropium/olodaterol (5/5 µg and 2.5/5 µg) and tiotropium (2.5 µg and 5 µg) and olodaterol (5 µg) monotherapy were compared.^22^ Change from baseline in FEV_1_ AUC_0–3_ response was significantly greater with both doses of tiotropium/olodaterol *versus* both doses of tiotropium or olodaterol monotherapy.^22^ There was a significant difference between both doses of tiotropium/olodaterol and tiotropium (2.5 µg) and olodaterol, but not tiotropium (5 µg) for change from baseline in trough FEV_1_ response.^22^ Furthermore, FEV_1_ AUC_0–3_ responses were better among patients classified as GOLD stage 2 than among those classified as GOLD stages 3–4, suggesting that patients with moderate airflow obstruction had a better response to tiotropium/olodaterol than those with more severe disease.^22^ In a prospective, multicenter, randomized, open-label, and parallel interventional study, 80 Japanese patients with treatment-naïve COPD were randomized to receive either tiotropium or tiotropium/olodaterol as a first-line treatment for 12 weeks.^48^ Change in FEV_1_ from baseline was significantly greater with tiotropium/olodaterol *versus* tiotropium monotherapy (mean difference: 138.7 mL; 95% CI: 52.8–224.6; *p* = 0.002).^48^ Changes in inspiratory capacity, forced vital capacity (FVC), and vital capacity were also higher with tiotropium/olodaterol than with tiotropium monotherapy; however, this was not statistically significant.^48^

In a prespecified analysis of the EMAX trial, change from baseline in trough FEV_1_ at week 24 was significantly greater with umeclidinium/vilanterol than with umeclidinium (mean difference: 44 mL; 95% CI: 1–87; *p* = 0.045) or salmeterol (mean difference: 128 mL; 95% CI: 85–171; *p* < 0.001).^47^ Mean change from baseline in trough FVC was significantly higher with umeclidinium/vilanterol versus umeclidinium (mean difference: 82 mL; 95% CI: 15–148; *p* = 0.016) and salmeterol (mean difference: 177 mL; 95% CI: 110–243; *p* < 0.001).^47^ There was a significant difference in mean change in trough inspiratory capacity when comparing umeclidinium/vilanterol and salmeterol (mean difference: 71 mL; 95% CI: 6–136; *p* = 0.032).^47^ In a post hoc analysis of pooled data from two large RCTs of umeclidinium/vilanterol, there was a significant difference in change from baseline in trough FEV1 (mL) in patients treated with umeclidinium/vilanterol versus umeclidinium (least square mean [95% CI] 60 [14–106]; *p* = 0.011) and versus vilanterol (72 [27–117]; *p* = 0.002]).^23^ In a post hoc pooled analysis of three RCTs of umeclidinium/vilanterol, the mean change from baseline in trough FEV_1_ at day 169 was higher in the maintenance therapy–naïve subgroup (mean difference: 146 mL; 95% CI: 102–189; *p* < 0.001) versus the intent-to-treat (ITT) population (mean difference: 95 mL; 95% CI: 71–118; *p* < 0.001).^24^

In a post hoc analysis of pooled data from the ARISE, SHINE, and SPARK trials, greater improvements were observed in the change from baseline in trough FEV_1_ with glycopyrronium/indacaterol versus tiotropium (treatment difference: 86 mL; 95% CI: 54–118) and glycopyrronium (treatment difference: 80 mL; 95% CI: 47–112) after 24/26 weeks of treatment.^25^ Furthermore, the proportion of patients achieving a minimal CID (MCID) in trough FEV_1_ (⩾100 mL improvement in trough FEV_1_) was significantly higher for glycopyrronium/indacaterol versus tiotropium (OR (95% CI): 1.97 (1.39–2.79)) and glycopyrronium (OR (95% CI): 2.24 [1.57–3.21]).^25^

In a post hoc analysis of the PINNACLE trials, glycopyrronium/formoterol showed a greater change from baseline in morning predose trough FEV_1_ versus formoterol (treatment difference: 56 mL; *p* < 0.0001) and glycopyrronium (treatment difference: 73 mL; *p* < 0.0001) at week 24.^26^ Similar improvements were observed with glycopyrronium/formoterol for the peak change from baseline in FEV_1_ within 2 h postdose versus formoterol (treatment difference: 79 mL) and glycopyrronium (treatment difference: 138 mL) at week 24.^26^ A subgroup analysis also revealed a better response with glycopyrronium/formoterol versus glycopyrronium and formoterol monotherapy in Chinese patients and symptomatic patients (CAT score: ⩾15).^26^

In the pooled analysis of the ACLIFORM and AUGMENT studies, patients treated with aclidinium/formoterol showed a significantly greater improvement from baseline in trough FEV_1_ at week 24 versus those treated with formoterol (treatment difference: 57 mL; *p* < 0.01) but not aclidinium (difference: 14 mL; *p* = 0.484).^27^ Significant improvements from baseline were noted in 1-h postdose FEV_1_ in patients treated with aclidinium/formoterol versus those treated with aclidinium (least-squares mean difference: 84 mL; *p* < 0.001) and formoterol (least-squares mean difference: 117 mL; *p* < 0.001; Table 3).^27^

**Table 3.** Efficacy of LAMA/LABA combination therapy versus LAMA or LABA monotherapy in maintenance therapy–naïve patients.

Study	Study design	Patient characteristics	LAMA/LABA	Lung function	COPD symptoms/QoL	Exacerbations/ rescue medication use/hospitalization	CID
Rabe et al. (2021)^28^	Post hoc analysis of pooled data from two RCTs (TOnado 1 and 2)	Outpatients aged ⩾40 years with a history of moderate to very severe COPD (GOLD stage 2–4); post-bronchodilator FEV_1_ <80% of predicted normal; post-bronchodilator FEV_1_/FVC < 70%; current or ex-smokers with a smoking history of > 10 pack–years^28^	T/O: 5/5 µg	T/O versus tiotropium: FEV_1_ responders^a^: 29.4% versus 44.5%	T/O versus tiotropium SGRQ responders^b^: 27.1% versus 31.7%	T/O versus tiotropium: Moderate or severe exacerbations: 19.4% versus 19.4%	T/O versus tiotropium Median time to event: 233 versus 171 days (HR: 0.75; 95% CI: 0.62–0.91; *p* < 0.0030)
Buhl et al. (2020)^20^	Post hoc analysis of pooled data from four RCTs (TOnado 1 and 2 and OTEMTO 1 and 2)	Not receiving LAMA, LABA, or ICS at trial enrollment	T/O: 5/5 µg	T/O versus tiotropium FEV_1_ responders^a^: 55.8% versus 41.1%	T/O versus tiotropium SGRQ responders^b^: 59.6% versus 48.8% TDI responders^c^: 63.3% versus 55.0%	NA	NA
Singh et al. (2016)^21^	Post hoc analysis of pooled data from two RCTs (OTEMTO 1 and 2)	No prior use of LAMA, LABA, and/or ICS	T/O: 5/5 µg	T/O showed significant improvement in FEV_1_ AUC_0–3_ but not trough FEV_1_ versus tiotropium	T/O showed significant improvement in SGRQ total score and TDI focal score versus tiotropium monotherapy	NA	NA
Ferguson et al. (2015)^22^	Post hoc analysis of pooled data from two RCTs (TOnado 1 and 2)	No prior LAMA or LABA treatment at baseline	T/O: 5/5 µg	GOLD 2 stage Adjusted mean (SE) T/O versus tiotropium FEV_1_ AUC_0–3_ (mL): 114 (19), *p* < 0.0001 Trough FEV_1_ (mL): 79 (20), *p* < 0.0001 T/O versus olodaterol FEV_1_ AUC_0–3_ (mL): 127 (19), *p* < 0.0001 Trough FEV_1_ (mL): 82 (20), *p* < 0.0001	NA	NA	NA
Takahashi et al. (2020)^48^	Prospective, multicenter, randomized, open-label, and parallel interventional study	Not treated with ICS, LABA, and/or LAMA in the last 12 months	T/O: 5/5 µg	Mean (95% CI) change from baseline (mL) T/O versus tiotropium FEV_1_: 139 (53 to 225), *p* = 0.002 IC: 115 (−36, 267), *p* = 0.13 FVC: 55 (−93 to 202), *p* = 0.46	TDI (points) Mean (95% CI) change from baseline T/O versus tiotropium: 0.9 (0.2 to 1.8), *p* = 0.02	NA	NA
Bjermer et al. (2021)^47^	Prespecified analysis of the EMAX trial	No use of maintenance bronchodilator (except for short-acting bronchodilators as rescue mediation) during the period from 30 days before screening until the first dose of study treatment	U/V: 62.5/25 µg	LS mean change (95% CI) from baseline (mL) U/V versus umeclidinium Trough FEV_1_: 44 (1 to 87), *p* = 0.045 Trough FVC: 82 (15 to 148), *p* = 0.016 Trough IC: 29 (−37 to 94), *p* = 0.388 U/V versus salmeterol Trough FEV_1_: 128 (85 to 171), *p* < 0.001 Trough FVC: 177 (110 to 243), *p* < 0.001 Trough IC: 71 (6 to 136), *p* = 0.032	SGRQ score at week 24 (OR [95% CI]) U/V versus umeclidinium 1.07 (0.75 to 1.53); *p* = 0.705 U/V versus salmeterol 1.39 (0.97 to 2.00); *p* = 0.072	Risk of a first moderate/severe exacerbation U/V versus umeclidinium HR (95% CI): 0.92 (−68 to 49) U/V versus salmeterol HR (95% CI): 0.58 (0 to 66) LS mean (95% CI) change from baseline in percent rescue medication-free days U/V versus umeclidinium 10.6 (4.9 to 16.3), *p* < 0.001 U/V versus salmeterol : 8.3 (2.6 to 14.0), *p* = 0.005	HR (95% CI) CID definition 1 (exacerbation, FEV_1_, SGRQ) U/V versus umeclidinium: 1.16 (0.88 to 1.52), *p* = 0.292 U/V versus salmeterol: 0.78 (0.60 to 1.00), *p* = 0.048 CID definition 2 (exacerbation, FEV_1_, CAT) U/V versus umeclidinium: 0.90 (0.69 to 1.18), *p* = 0.454 U/V versus salmeterol: 0.70 (0.54 to 0.90), *p* = 0.006 CID definition 3 (exacerbation, SGRQ, CAT, SAC-TDI) U/V versus umeclidinium: 0.84 (0.67 to 1.06), *p* = 0.144 U/V versus salmeterol: 0.80 (0.64 to 1.00), *p* = 0.052
Slade et al. (2021)^52^	Retrospective matched cohort study using healthcare insurance claims from Optum’s de-identified Clinformatics Data Mart database	Initiating maintenance therapy with LAMA/LABA or LAMA monotherapy	U/V	NA	NA	U/V versus tiotropium: COPD-related inpatient admission: 24.1% versus 26.1%; HR (95% CI): 0.87 (0.79 to 0.96); *p* = 0.006 Mean time to first COPD-related admission (days): 88 versus 66	NA
Naya et al. (2019)^23^	Post hoc analysis of pooled data from two large RCTs (NCT01313637, NCT01313650)	No COPD medication, except short-acting bronchodilators used as rescue medication recorded in the 30 days before screening	U/V: 125/25 µg U/V: 62.5/25 µg	LS mean (95% CI) change from baseline in trough FEV_1_ (mL) U/V versus umeclidinium: 60 (14 to 106), *p* = 0.011 U/V versus vilanterol: 72 (27 to 117), *p* = 0.002	NA	Percent risk reduction for a first moderate/severe exacerbation U/V versus umeclidinium: 51 (−8 to 77), *p* = 0.076 U/V versus vilanterol: 60 (16 to 81), *p* = 0.016	NA
Maleki-Yazdi et al. (2017)^24^	Post hoc pooled analysis of three RCTs (NCT01777334, NCT01316913, and NCT01316900)	Receiving no maintenance therapy for ⩾30 days before screening	U/V: 62.5/25 µg and U/V: 125/25 µg	LS mean change (95% CI) from baseline in trough FEV_1_ (mL) U/V versus tiotropium: 146 (102 to 189), *p* < 0.001	SGRQ responders^b^; OR (95% CI) U/V versus tiotropium Day 28: 58% versus 55%; 1.2 (0.8 to 1.7) Day 84: 56% versus 53%; 1.1 (0.8 to 1.6) Day 168: 54% versus 50%; 1.2 (0.8 to 1.7)	Patients achieving a response in rescue-free episodes U/V versus tiotropium: 47% versus 37%; OR (95% CI): 1.5 (1.0 to 2.2)	Risk of a first CID HR (95% CI): U/V versus tiotropium: 0.66 (0.51 to 0.85), *p* = 0.001
Muro et al., 2020^25^	Post hoc pooled analysis of three phase III RCTs (ARISE, SHINE, and SPARK)	Patients who were not on maintenance treatment with a LABA, LAMA, LABA/ICS, or LABA/ICS+LAMA at baseline/study entry	G/I: 50/110 µg	Change (95% CI) from baseline in trough FEV_1_ (mL): G/I versus tiotropium: 86 (54 to 118) G/I versus glycopyrronium: 80 (47 to 112)	Mean change (95% CI) from baseline G/I versus tiotropium SGRQ total score: −1.808 (−3.783 to 0.168) TDI focal score: 0.634 (−0.012 to 1.281 G/I versus glycopyrronium SGRQ total score: −0.809 (−2.829 to 1.210) TDI focal score: 0.286 (−0.345 to 0.918)	Mean change (95% CI) from baseline in the number of puffs/day G/I versus tiotropium −0.531 (−0.869 to −0.192) G/I versus glycopyrronium: −0.499 (−0.849 to −0.150)	NA
Zheng et al., 2020^26^	Post hoc pooled analysis of three phase III RCTs (PINNACLE-1, -2, and -4)	Received short-acting bronchodilators or remained untreated	G/F: 14.4/9.6 µg	LS mean change from baseline G/F versus formoterol Morning predose trough FEV_1_ 56 mL, *p* < 0.0001 FEV_1_ within 2 h postdose: 79 mL, *p* < 0.0001 G/F versus glycopyrronium Morning predose trough FEV_1_: 73 mL *p* < 0.0001 FEV_1_ within 2 h postdose: 138 mL, *p* < 0.0001	Mean change from baseline treatment difference (95% CI) G/F versus formoterol SGRQ total score: 0.81 (−0.69 to 2.31), *p* = 0.2903 G/F versusglycopyrronium SGRQ total score: −0.39 (−1.92 to 1.14), *p* = 0.6179	Mean change (95% CI) from baseline in rescue medication use G/F versus formoterol: −0.2 (−0.5 to 0.2), *p* = 0.3845 G/F versus glycopyrronium: −0.2 (−0.6 to 0.2), *p* = 0.2740	Risk reduction G/F versus formoterol: 17%, *p* = 0.0157 G/F versus glycopyrronium: 21%, *p* = 0.0018
Singh et al., 2019^27^	Post hoc subgroup analysis of two phase III RCTs (ACLIFORM and AUGMENT)	Not received prior maintenance therapy for COPD: LABA, LAMA, ICS, systemic corticosteroids, or xanthines; short-acting bronchodilators were permitted	A/F: 400/12 µg	LS mean difference from baseline A/F versus aclidinium Tough FEV_1_: 14 mL, *p* = 0.484 1 h postdose FEV_1_: 84 mL, *p* < 0.001 A/F versus formoterol Tough FEV_1_: 57 mL, *p* < 0.01 1 h postdose FEV_1_: 117 mL (*p* < 0.001)	Change from baseline A/F versus aclidinium SGRQ total score: −3.1, *p* < 0.01 TDI focal score: 1.17, *p* < 0.001) E-RS total score: −0.82, *p* < 0.05 A/F versus formoterol SGRQ total score: −2.3, *p* < 0.05 TDI focal score: 0.92, *p* < 0.01 E-RS total score: −0.83, *p* < 0.05	NA	NA

a ⩾100-mL improvement from baseline; ^b^⩾4-unit improvement from baseline; ^c^⩾1-unit improvement from baseline.

A/F, aclidinium/formoterol; AUC_0–3_, area under the curve from 0 to 3 h; CI, confidence interval; CID, clinically important deterioration; COPD, chronic obstructive pulmonary disease; E-RS, Evaluating Respiratory Symptoms; FEV_1_, forced expiratory volume in 1 s; FVC, forced vital capacity; G/F, glycopyrronium/formoterol; G/I, glycopyrronium/indole; HR, hazard ratio; IC, inspiratory capacity; ICS, inhaled corticosteroid; LABA, long-acting β_2_-agonist; LAMA, long-acting muscarinic antagonist; LS, least squares; NA, not available; OR, odds ratio; QoL, quality of life; RCT, randomized controlled trial; SE, standard error; SGRQ, St. George’s Respiratory Questionnaire; TDI, Transitional Dyspnea Index; T/O, tiotropium/olodaterol; U/V, umeclidinium/vilanterol.

### PROs

PRO measures in COPD can be broadly categorized as those based on health-related QoL (e.g., SGRQ total score and CAT) and those based on symptoms (e.g., transition dyspnea index (TDI) focal score, self-administered computerized TDI [SAC-TDI] focal score, and E-RS COPD total score).^49^ SGRQ is the most widely used and US Food and Drug Administration–qualified COPD-specific instrument that has 50 items comprising three domains: symptoms, activity, and impact.^49^ CAT is an eight-item unidimensional measure of health status impairment in COPD.^50^ TDI is a three-item interview-based measure that captures the effect of dyspnea along with the effect on activities of daily living.^49^ SAC-TDI is a self-administered computerized version of TDI that captures breathlessness related to daily activities.^49^ E-RS is an 11-item daily diary that quantifies the severity of respiratory symptoms.^49^

### SGRQ total score

A change of at least 4 units from the baseline SGRQ total score is considered clinically meaningful.^20^ In a pooled analysis of TOnado 1 and 2 and OTEMTO 1 and 2, both tiotropium/olodaterol and tiotropium provided clinically relevant improvements in the SGRQ total score after 12 weeks of treatment (mean difference: −1.780 ± 0.686; 95% CI: −3.126 to −0.434; *p* = 0.0096).^20^ A subgroup analysis of SGRQ total score change from baseline at week 12 revealed a significant improvement in patients with moderate COPD and those with BDI ⩽6.^20^ In a post hoc analysis of the OTEMTO studies, 52.8% of patients in the GOLD 2 subgroup and 51.7% in the GOLD 3 subgroup were SGRQ responders among those treated with tiotropium/olodaterol (5/5 µg).^21^ An improvement in SGRQ total score with tiotropium/olodaterol versus tiotropium was also reported in another post hoc analysis of pooled data from TOnado 1 and 2 and OTEMTO 1 and 2.^21,28^ In a post hoc pooled analysis of three RCTs of umeclidinium/vilanterol, the mean change from baseline in the SGRQ total score favored umeclidinium/vilanterol in the maintenance therapy–naïve subgroup at all time points compared with the tiotropium maintenance therapy–naïve subgroup, umeclidinium/vilanterol ITT, and tiotropium ITT populations; however, this difference was not statistically significant.^24^ In a prespecified analysis of the EMAX trial, the proportion of responders for SGRQ total score at week 24 was not significantly different among umeclidinium/vilanterol (47%), umeclidinium (46%), and salmeterol (40%).^47^ In the post hoc pooled analysis of the ARISE, SHINE, and SPARK trials, the change from baseline in the SGRQ total score was numerically greater with glycopyrronium/indacaterol than with tiotropium and glycopyrronium.^25^ Moreover, a higher proportion of patients treated with glycopyrronium/indacaterol achieved the MCID for the SGRQ total score than those treated with tiotropium (66.6% versus 60.6%) and glycopyrronium (66.6% versus 64.6%).^25^ In a post hoc pooled analysis of PINNACLE-1, -2, and -4, there was no significant difference in mean change from baseline treatment difference in SGRQ total score.^26^ In the post hoc analysis of the ACLIFORM and AUGMENT studies, a significant improvement from baseline in the SGRQ total score was noted at week 24 for aclidinium/formoterol combination therapy versus aclidinium and formoterol monotherapy.^27^ Furthermore, while the aclidinium/formoterol, aclidinium, and formoterol groups exceeded the MCID versus baseline, only aclidinium/formoterol exceeded the MCID versus placebo (Table 3).^27^

### CAT score

In a prespecified analysis of the EMAX trial in maintenance therapy–naïve patients, the mean change from baseline in the CAT score was not significantly different among umeclidinium/vilanterol, umeclidinium, and salmeterol (Table 3).^47^

### TDI focal score

The MCID for the TDI focal score was considered as a ⩾1-point improvement in the score.^25^ In a pooled analysis of TOnado 1 and 2 and OTEMTO 1 and 2, tiotropium/olodaterol significantly improved TDI score after 12 weeks of treatment compared with tiotropium (treatment difference, mean ± SE: 0.409 ± 0.169; 95% CI: 0.077–0.741; *p* = 0.0158).^20^ Furthermore, in a subgroup analysis of TOnado 1 and 2 and OTEMTO 1 and 2, patients with moderate COPD, SGRQ score at baseline equal to or greater than the median value, and BDI score > 6 at baseline showed significantly greater improvements in the TDI score with tiotropium/olodaterol than with tiotropium at week 12.^20^ In the post hoc analysis of the OTEMTO trials, the treatment difference in the posttreatment TDI focal score was significantly greater with tiotropium/olodaterol versus tiotropium.^21^

In an integrated post hoc intent-to-treat analysis of two RCTs, no significant difference was noted between umeclidinium/vilanterol versus placebo in the TDI focal score at day 168 in the maintenance therapy–naïve subgroup (difference: 0.9; 95% CI: 0.3–1.5).^23^ In a prospective, multicenter, randomized, open-label, parallel study, tiotropium/olodaterol maintenance therapy–naïve Japanese patients had significantly improved TDI focal score after 12 weeks compared with tiotropium monotherapy (mean difference: 0.9; 95% CI: 0.2–1.8; *p* = 0.02).^48^ In the pooled analysis of the ARISE, SHINE, and SPARK trials, the change from baseline in the TDI focal score was greater with glycopyrronium/indacaterol versus tiotropium and glycopyrronium.^25^ Furthermore, a higher proportion of patients treated with glycopyrronium/indacaterol achieved the MCID for the TDI focal score than those treated with tiotropium (74.1% versus 66.4%) and glycopyrronium (74.1% versus 71.8%).^25^ In a post hoc analysis of ACLIFORM and AUGMENT, the TDI focal scores at week 24 were significantly improved in patients who were treated with aclidinium/formoterol versus those who were treated with aclidinium (least square mean difference: 1.17; *p* < 0.001) and formoterol (0.92; *p* < 0.01; Table 3).^27^

### E-RS total score and SAC-TDI score

In the prespecified analysis of the EMAX trial, greater mean improvements from baseline in the E-RS total score and SAC-TDI focal score were observed with umeclidinium/vilanterol versus umeclidinium or salmeterol at all time points in both the maintenance therapy–naïve and maintenance treatment subgroups.^47^ In the pooled post hoc analysis of the ACLIFORM and AUGMENT trials, aclidinium/formoterol showed significant improvements in the E-RS total score versus aclidinium and formoterol and early morning and nighttime symptom severity versus aclidinium (Table 3).^27^

### Rescue medication-free days

Mean improvements in rescue medication inhalations/day and the proportion of rescue medication-free days were significantly greater with umeclidinium/vilanterol versus both monotherapies at all time points and over weeks 1–24 in the prespecified analysis of the EMAX trial.^47^ In the pooled analysis of three RCTs of umeclidinium/vilanterol, a higher proportion of patients treated with umeclidinium/vilanterol achieved a response in rescue-free episodes than those treated with tiotropium (47% versus 37%; odds ratio: 1.5; 95% CI: 1.0–2.2; *p* < 0.05). Umeclidinium/vilanterol also reduced the mean number of puffs per day over the study period (difference: −0.5; 95% CI: −0.9 to 0.0; *p* = 0.066).^24^ In the pooled analysis of the ARISE, SHINE, and SPARK trials, the change from baseline in the mean daily number of puffs was significantly greater with glycopyrronium/indacaterol than with tiotropium and glycopyrronium (Table 3).^25^

### Exacerbations

In the EMAX prospective analysis, exacerbation risk was similar between the umeclidinium/vilanterol and umeclidinium groups in maintenance therapy–naïve patients.^47^ In a pooled analysis of RCTs of umeclidinium/vilanterol, the maintenance therapy–naïve subgroup had a lower incidence of exacerbations than the maintenance treatment subgroup.^23^

### Composite endpoint: CID

As COPD is a multidimensional disease, composite endpoints, including a measure of CID, which encompasses lung function, health status, and the occurrence of moderate-to-severe exacerbations, have been used in clinical studies, proving to be a valuable measure of disease progression in COPD.^51^ According to the subgroup analysis of the EMAX study, reduced risk of a first CID was observed with umeclidinium/vilanterol versus salmeterol but not umeclidinium for all three definitions (Table 3 and Supplemental Table 1).^47^ In a responder analysis using a logistic regression model with treatment and study as covariates, the proportion of patients classified as FEV_1_ (⩾0.1-L improvement), SGRQ (⩾4-unit improvement), or TDI (⩾1-unit improvement) responders was higher with tiotropium olodaterol versus tiotropium (FEV_1_: 55.8% versus 41.1%, SGRQ: 59.6% versus 48.8%, and TDI: 63.3% versus 55.0%).^20^ In the post hoc analysis of pooled data from TOnado 1 and 2, there was a 25% reduced risk in the median time to first CID in the tiotropium/olodaterol group versus the tiotropium group (HR [95% CI]: 0.75 [0.62–0.91]; *p* < 0.0030).^28^ In the pooled analysis of TOnado 1 and 2 and OTEMTO 1 and 2, tiotropium/olodaterol increased the odds of achieving an MCID by 80.7% for trough FEV_1_, 54.4% for SGRQ total score, and 43.3% for TDI focal score, compared with tiotropium.^20^ Patients treated with tiotropium/olodaterol were 60% more likely to experience an MCID in trough FEV_1_, SGRQ score, or TDI score after 12 weeks than those treated with tiotropium alone.^20^ In a post hoc pooled analysis of three RCTs of umeclidinium/vilanterol, the risk of a first CID was significantly lower with umeclidinium/vilanterol versus umeclidinium (HR [95% CI: 0.66 [0.51–0.85]; *p* = 0.001].^24^ A subgroup analysis of the PINNACLE trials revealed significant improvements in patients with severe COPD, a lower BDI score, and greater symptom burden at baseline. The number of patients with CID was lower with glycopyrronium/formoterol than with formoterol (54.7% versus 60.4%) and glycopyrronium (54.7% versus 62.6%). The median time to first CID was significantly longer with glycopyrronium/formoterol versus formoterol (20.1 weeks versus 16.1 weeks).^26^ Furthermore, glycopyrronium/formoterol reduced the risk of CID by 21% versus glycopyrronium (hazard ratio: 0.79; 95% CI: 0.67–0.91; *p* = 0.0018) and 17% versus formoterol (hazard ratio: 0.83; 95% CI: 0.71–0.97; *p* = 0.0157; Table 3).^26^

### Safety and caveats around post hoc analyses of RCTs

In the pooled analysis of TOnado 1 and 2 and OTEMTO 1 and 2,^20^ the post hoc analysis of TOnado 1 and 2,^28^ the prespecified analysis of the EMAX trial,^47^ and the post hoc analysis of the PINNACLE trials,^26^ the proportion of reported adverse events was similar for LAMA/LABA combination therapy and monotherapy in the maintenance therapy–naïve population. No safety evaluations were reported for the maintenance therapy–naïve patients in the pooled analysis of the ARISE, SHINE, and SPARK trials^25^ and that of the ACLIFORM and AUGMENT trials.^27^ Thus, LAMA/LABA combination therapy in maintenance therapy–naïve patients showed greater benefits than monotherapy, without compromising patient safety.

There are some limitations in the post hoc analyses of the pooled data from RCTs that were used in the present review to extrapolate outcomes. The definition of maintenance therapy–naïve patients was not consistent across the included studies; for example, it was defined as no prior use of LAMAs, LABAs, and/or ICS in the pooled analysis of the OTEMTO 1 and 2 trials^21^; the other definition for maintenance therapy–naïve patients included those not receiving maintenance treatment (LABA, LAMA, LABA/ICS, or LABA/ICS+LAMA) at screening or study entry (pooled analysis of the ARISE, SHINE, and SPARK trials);^25^ those who received short-acting β_2_-agonists (SABAs) or were untreated (pooled analysis of the PINNACLE trials)^26^; and those not receiving maintenance therapy for ⩾30 days before screening (pooled analysis of three RCTs of umeclidinium/vilanterol).^24^ In the pooled analysis of the ARISE, SHINE, and SPARK trials, exacerbation rates were analyzed only in the SPARK study; therefore, the effect of LAMA/LABA on exacerbation rates could not be assessed.^25^ Hence, there might be an underestimation of the effect of the LAMA/LABA combination therapy on the exacerbation rate in maintenance therapy–naïve patients.^25^ Some pooled analyses used doses of LAMA and LABA FDCs that are not approved in the US (e.g., umeclidinium/vilanterol: 125/25 µg, whereas the approved dose is 62.5/25 µg).^13,23,24^ The inhaler device used for the delivery of LAMA or LABA was not the same as that approved for the treatment of patients with COPD (e.g., comparison of tiotropium monotherapy with HandiHaler^®^ in umeclidinium/vilanterol trials, although the approved inhaler for tiotropium delivery is Respimat^®^).^24^ Thus, extrapolation of the clinical relevance of the findings from this pooled analysis is questionable. There is a need for controlled studies evaluating the effect of LAMA/LABA combination therapy in maintenance therapy–naïve patients.

## Real-world studies of LAMA/LABA combination therapy in maintenance therapy–naïve patients with COPD

A few real-world studies have explored LAMA/LABA combination therapy in maintenance therapy–naïve patients with COPD. A retrospective matched cohort study conducted between January 1, 2013 and December 31, 2018 evaluated the risk of admissions and readmissions in patients with COPD receiving initial maintenance therapy with umeclidinium/vilanterol or tiotropium.^52^ Over 12 months, patients initiating umeclidinium/vilanterol had a significantly reduced risk of COPD-related inpatient admissions (hazard ratio: 0.87; 95% CI: 0.79–0.96; *p* = 0.006) and rate of on-treatment COPD-related inpatient admissions (rate ratio: 0.80; 95% CI: 0.72–0.92; *p* = 0.008) than those initiating tiotropium. While all-cause readmission rates were similar between the treatment cohorts, all-cause inpatient readmission rates among patients with an initial admission length of stay of 1–3 days were numerically lower for umeclidinium/vilanterol versus tiotropium (30-day readmissions: 10.5% versus 12.4%; 90-day readmissions: 15.5% versus 19.8%). Similar patterns were observed for COPD-related readmissions.^52^ In a post hoc analysis of a 52-week postmarketing surveillance study to assess the effectiveness of tiotropium/olodaterol, CAT score and lung function improvement were greatest in treatment-naïve patients: mean total CAT score of −7.6 (95% CI: −9.2 to −6.1), mean FEV_1_ of 0.177 L (95% CI: 0.076–0.279), and mean FVC of 0.178 L (95% CI: 0.036–0.319); however, there was no comparator arm in this study.^53^ A 6-week, open-label, single-arm, noninterventional study analyzed the potential changes in clinical control using the Clinical COPD Questionnaire (CCQ) score in patients with COPD (maintenance therapy–naïve: receiving LAMA or LABA monotherapy or ICS/LABA at baseline) receiving tiotropium/olodaterol in routine clinical practice.^54^ Overall, of the 4700 study participants, 81.4% (95% CI: 80.24–82.49) of patients achieved therapeutic success after 6 weeks of treatment, regardless of the treatment pathway. An improved CCQ score was seen in 92.2% of patients.^54^ The greatest benefit was seen in maintenance therapy–naïve patients (n = 2,678; therapeutic success: 85.7%; 95% CI: 84.28–86.97; nominal *p* < 0.0001).^54^ However, these results need to be interpreted with caution considering that the study was not randomized or controlled. The findings may be attributed to the placebo effect, regression to the mean, and other uncontrolled patient or treating physician factors. In the Polish subgroup of this study, 72.4% of patients achieved therapeutic success after 6 weeks of treatment, which was more pronounced in the treatment-naïve group (83.4%) versus those previously treated with a LAMA (62.6%) or a LABA (73.3%).^55^

A prospective, open-label, noninterventional study in patients with COPD receiving tiotropium/olodaterol found that treatment-naïve patients had a higher therapeutic success rate (defined as a 10-point increase in the Physical Functioning Questionnaire between baseline and weeks 4–6) than those who received prior maintenance therapy (59.1% versus 44.5%; *p* < 0.0001).^56^ These differences were driven by a higher response in treatment-naïve patients who were classified as GOLD B (59.8%) and C (63.0%), whereas the proportion of patients achieving therapeutic success was similar for GOLD D patients, regardless of a previous history of maintenance treatment.^56^

In the noninterventional DACCORD study conducted in Germany, the majority of patients who were naïve to glycopyrronium/indacaterol did not experience any exacerbations during the 6 months before recruitment and during the 1-year follow-up.^57^ Furthermore, the annualized exacerbation rate during the 1-year follow-up period was 0.16 (95% CI: 0.12–0.21) in the overall population and 0.35 (95% CI: 0.23–0.53) in the subgroup of patients who experienced exacerbations in the 6 months before recruitment.^57^ Additionally, the mean CAT total score improved at 3 and 12 months, exceeding the clinically relevant difference (2 units) at both timepoints.^57^ Approximately 55% and > 60% of patients reported a clinically relevant improvement at 3 months and 12 months, respectively.^57^

## Expert recommendations

**Figure 1.**
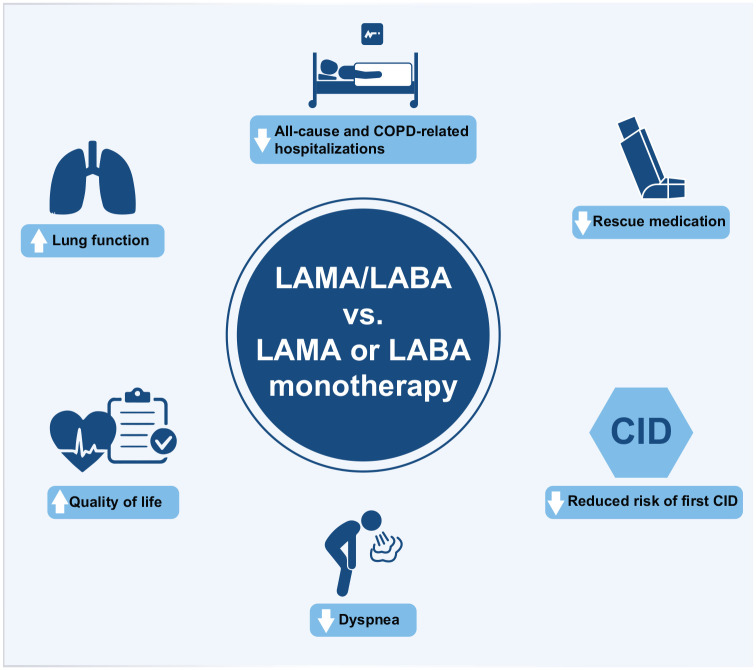
Potential benefits of LAMA/LABA combination therapy compared to LAMA or LABA monotherapy in patients with COPD. CID, clinically important deterioration; COPD, chronic obstructive pulmonary disease; LABA, long-acting β_2_-agonist; LAMA, long-acting muscarinic antagonist.

As the fastest decline in lung function occurs in the initial stages of COPD, adequate treatment in the early stages of the disease could help patients achieve more rapid control of respiratory symptoms and prevent the downward spiral triggered by the loss of lung function and consequent physical deconditioning. Evidence from clinical trials suggests that treatment with LAMA/LABA combination therapy provides greater improvements in, but not limited to, trough FEV1 and PROs such as a reduction in rescue medication use, compared to LAMA or LABA monotherapy in maintenance treatment-naïve patients with COPD (Figure 1). Early initiation of LAMA/LABA combination therapy may reduce the risk of short-term deterioration compared with monotherapy in symptomatic patients with COPD and is also recommended by the GOLD 2024 report, 2023 CTS COPD guidelines, 2020 ATS Clinical Practice Guideline, Spanish COPD guidelines, and UK NICE guidelines. Composite endpoints, such as CID, could be a valuable metric for monitoring COPD progression and assessing therapeutic effects in future randomized COPD trials.

## Conclusion

As there is a rapid and irreversible decline in lung function early in the course of COPD, it is critical that patients receive effective clinical management to preserve lung function, improve QoL, and prevent CID. The overall evidence presented in this review supports the use of LAMA/LABA combination therapy as a first-line treatment in maintenance therapy–naïve patients with moderate COPD symptoms (Supplemental Figure 1). Collectively, data from previous studies on LAMA/LABA combination therapy versus monotherapy suggest that early treatment with LAMA/LABA may be more effective than that with LAMA or LABA monotherapy in the early management of COPD. This recommendation for prescribing LAMA/LABA combination therapy to maintenance therapy–naïve patients with COPD is in accordance with patient-centric guidelines and necessitates greater adherence to guideline-directed therapy to improve outcomes.

## Supplemental Material

sj-docx-1-tar-10.1177_17534666241279115 – Supplemental material for Long-acting muscarinic antagonist and long-acting β2-agonist combination for the treatment of maintenance therapy–naïve patients with chronic obstructive pulmonary disease: a narrative review Supplemental material, sj-docx-1-tar-10.1177_17534666241279115 for Long-acting muscarinic antagonist and long-acting β2-agonist combination for the treatment of maintenance therapy–naïve patients with chronic obstructive pulmonary disease: a narrative review by Roland Buhl, Marc Miravitlles, Antonio Anzueto and Stephen Brunton in Therapeutic Advances in Respiratory Disease

sj-pdf-2-tar-10.1177_17534666241279115 – Supplemental material for Long-acting muscarinic antagonist and long-acting β2-agonist combination for the treatment of maintenance therapy–naïve patients with chronic obstructive pulmonary disease: a narrative review Supplemental material, sj-pdf-2-tar-10.1177_17534666241279115 for Long-acting muscarinic antagonist and long-acting β2-agonist combination for the treatment of maintenance therapy–naïve patients with chronic obstructive pulmonary disease: a narrative review by Roland Buhl, Marc Miravitlles, Antonio Anzueto and Stephen Brunton in Therapeutic Advances in Respiratory Disease

## Declarations

**Ethics approval and consent to participate:** Not applicable.

**Consent for publication:** Not applicable.

**Competing interests:** RB reports grants and personal fees from AstraZeneca, Berlin-Chemie, Boehringer Ingelheim, Chiesi, Cipla, GlaxoSmithKline, Novartis, Roche, and Teva. MM has received speaker fees from AstraZeneca, Boehringer Ingelheim, Chiesi, Cipla, Menarini, Rovi, Bial, Kamada, Sandoz, Zambon, CSL Behring, Grifols, and Novartis; consulting fees from AstraZeneca, Atriva Therapeutics, Boehringer Ingelheim, Chiesi, GlaxoSmithKline, Bial, Gebro Pharma, CSL Behring, Inhibrx, Laboratorios Esteve, Ferrer, Mereo Biopharma, Verona Pharma, Spin Therapeutics, ONO Pharma, pH Pharma, Palobiofarma SL, Takeda, Novartis, Sanofi, and Grifols; and research grants from Grifols. AA reports speaking fees from Boehringer Ingelheim, AstraZeneca, Grifols, and Novartis, and consulting fees from Boehringer Ingelheim, GlaxoSmithKline, Sunovion, Novartis, and Grifols. SB has served on the advisory boards of AstraZeneca and Mylan and has been a speaker for AstraZeneca.

**Availability of data and materials:** Data sharing is not applicable to this article, as no datasets were generated or analyzed during the current study. The data included in this review are all sourced from published information and are publicly available.
