# Supplementary material for: Long-acting muscarinic antagonist and long-acting β2-agonist combination for the treatment of maintenance therapy–naïve patients with chronic obstructive pulmonary disease: a narrative review
Source: Ther Adv Respir Dis. 2024 Oct 1;18:17534666241279115. doi: 10.1177/17534666241279115 (PMC11456191; doi:10.1177/17534666241279115)
Supplement: sj-docx-1-tar-10.1177_17534666241279115 – Supplemental material for Long-acting muscarinic antagonist and long-acting β2-agonist combination for the treatment of maintenance therapy–naïve patients with chronic obstructive pulmonary disease: a narrative review [file sj-docx-1-tar-10.1177_17534666241279115.docx]

**Supplementary Table 1. CID definitions according to Bjermer *et al*.^47^**

| **Definition** | **Characteristics** |
| --- | --- |
| **1** | A first moderate or severe exacerbation and/or a decrease in trough FEV_1_ from baseline of ≥100 mL and/or a deterioration in health status indicated by an increase in the SGRQ score from baseline of ≥4 units. |
| **2** | A first moderate or severe exacerbation and/or a decrease in trough FEV_1_ from baseline of ≥ 100 mL and/or a deterioration in health status indicated by an increase in the CAT™ score from baseline of ≥ 2 units. |
| **3** | A first moderate or severe exacerbation and/or an SGRQ deterioration and/or a CAT™ deterioration, and/or an SAC-TDI deterioration indicated by a decrease of ≥1 unit. |

CAT^TM^, COPD Assessment Test; CID, clinically important deterioration; FEV_1_, forced expiratory volume in 1 s; SAC-TDI, self-administered computerized-Transition Dyspnea Index; SGRQ, St. George’s Respiratory Questionnaire.
