# Supplementary material for: Long-acting muscarinic antagonist and long-acting β2-agonist combination for the treatment of maintenance therapy–naïve patients with chronic obstructive pulmonary disease: a narrative review
Source: Ther Adv Respir Dis. 2024 Oct 1;18:17534666241279115. doi: 10.1177/17534666241279115 (PMC11456191; doi:10.1177/17534666241279115)
Supplement: sj-pdf-2-tar-10.1177_17534666241279115 – Supplemental material for Long-acting muscarinic antagonist and long-acting β2-agonist combination for the treatment of maintenance therapy–naïve patients with chronic obstructive pulmonary disease: a narrative review [file sj-pdf-2-tar-10.1177_17534666241279115.pdf]

LONG-ACTING MUSCARINIC ANTAGONIST AND LONG-ACTING β_2-AGONIST COMBINATION FOR THE TREATMENT OF MAINTENANCE THERAPY–NAÏVE PATIENTS WITH CHRONIC OBSTRUCTIVE PULMONARY DISEASE: A NARRATIVE REVIEW

Buhl R et al. *Therapeutic Advances in Respiratory Disease*

BACKGROUND:

COPD is a leading cause of morbidity and mortality worldwide.[1]

Rapid and irreversible lung function decline occurs early in the disease,[2-4] highlighting the critical need for effective clinical management.

Long-acting bronchodilators (LAMA and LABA) are the gold standard for COPD management.[5]

Combining a LAMA with a LABA helps achieve better clinical outcomes in patients who are not effectively controlled with a single long-acting bronchodilator.[6,7]

Single-inhaler therapies containing a fixed-dose combination of a LAMA and a LABA are commercially available for the maintenance treatment of patients with COPD: tiotropium/olodaterol, umeclidinium/vilanterol, glycopyrronium/indacaterol, glycopyrronium/formoterol, and aclidinium/formoterol.[8-12]

RATIONALE:

We reviewed the rationale for early initiation of LAMA/LABA combination therapy in maintenance therapy–naïve patients with COPD.

LITERATURE REVIEW:

There is no high-quality evidence such as that from RCTs to support initial pharmacological treatment strategies in newly diagnosed patients with COPD.[5] Evidence for the use of LAMA/LABA combination therapy in maintenance therapy–naïve patients with COPD are mostly based on post hoc analyses of pooled data from RCTs[13-21] and one pre-specified analysis of a RCT.[22]

KEY FINDINGS:

LAMA/LABA combination therapy versus monotherapy provided

Greater improvements in

- Lung function[13-15,17-22]
- Symptoms[13,14,16,18,20,22]
- Health-related QoL[13,14,17,18,20,22]

Reduced risk of

- Rescue medication use[17,18,22]
- First moderate/severe exacerbation[16,22]
- First CID*[13,19,21,22]

Both LAMA/LABA combination therapy and individual LAMA and LABA monotherapies have a **similar safety profile**.[13,19,21,22]

LAMA/LABA combination therapy may be more effective than monotherapy in the **early management** of COPD.

*Exacerbations, Lung function, Health-related QoL

CONCLUSION:

LAMA/LABA combination therapy may be more useful than LAMA or LABA monotherapy as a first-line treatment in maintenance therapy–naïve patients with moderate COPD symptoms.

Abbreviations: CID, clinically important deterioration; **COPD**, chronic obstructive pulmonary disease; **LABA**, long-acting β_2-agonist; **LAMA**, long-acting muscarinic antagonist; **QoL**, quality of life; **RCT**, randomized controlled trial.

References: 1. Chen S, et al. *Lancet Glob Health* 2023; 11: e1183–e1193 **2**. Young AL, et al. *Am J Respir Crit Care Med* 2020; 201: 294–302. **3**.Tantucci C and Modina D. *Int J Chron Obstruct Pulmon Dis* 2012; 7: 95–99. **4**. Rennard SI and Drummond MB. *Lancet* 2015; 385: 1778–1788. **5**. Global initiative for chronic obstructive lung disease. Global strategy for the diagnosis, management, and prevention of chronic obstructive pulmonary disease (2024 report), https://goldcopd.org/wp-content/uploads/2023/12/GOLD-2024_v1.1-1Dec2023_WMV.pdf 2023, accessed 23 January 2024 **6**. Miravitlles M, et al. *J Clin Med* 2022; 11: 6623. **7**. Singh D, et al. *Eur Respir Rev* 2021; 30: 210023. **8**. Boehringer Ingelheim. Prescribing information for STIOLTO RESPIMAT, https://www.accessdata.fda.gov/drugsatfda_docs/label/2019/206756s011lbl.pdf (2019, accessed 29 March 2023). **9**. GlaxoSmithKline. Prescribing information for ANORO ELLIPTA, https://www.accessdata.fda.gov/drugsatfda_docs/label/2022/203975s013lbl.pdf (2022, accessed 29 March 2023). **10**. Sunovion Pharmaceuticals Inc. Prescribing information for UTIBRON NEOHALER, https://www.accessdata.fda.gov/drugsatfda_docs/label/2019/207930s004lbl.pdf (2019, accessed 29 March 2023). **11**. AstraZeneca. Prescribing information fro BEVESPI AEROSPHERE, https://www.accessdata.fda.gov/drugsatfda_docs/label/2020/208294s009lbl.pdf (2019, accessed 29 March 2023). **12**. Circassia Pharmaceuticals Inc. Prescribing information for DUAKLIR PRESSAIR, https://www.accessdata.fda.gov/drugsatfda_docs/label/2019/210595lbl.pdf (2019, accessed 29 March 2023). **13**. Buhl R, et al. *Adv Ther* 2020; 37: 4175-4189 **14**. Singh D, et al. *Respir Res* 2016; 17: 73. **15**. Ferguson GT, et al. *Adv Ther* 2015; 32: 523-536. **16**. Naya I, et al. *Respir Res* 2019; 20: 60. **17**. Maleki-Yazdi MR, et al. *Adv Ther* 2017; 33: 2188-2199. **18**. Muro S, et al. *Respirology* 2020; 25: 393-400. **19**. Zheng J, et al. *Respir Res* 2020; 21: 69. **20**. Singh D, et al. *Int J Chron Obstruct Pulmon Dis* 2019; 14: 2835-2848. **21**. Rabe KF, et al. *Adv Ther* 2021; 38: 579-593. **22**. Bjermer L, et al. *Int J Chron Obstruct Pulmon Dis* 2021; 16: 1939–1956.
